# On-Admission Anemia and Survival Rate in COVID-19 Patients

**DOI:** 10.52547/ibj.3703

**Published:** 2022-10-29

**Authors:** Reza Asadzadeh, Aliashraf Mozafari, Elham Shafiei, Mohammadreza Kaffashian, Iraj Ahmadi, Mohammadzaman Darvish, Saiyad Bastaminejad

**Affiliations:** 1 School of Medicine, Ilam University of Medical sciences, Ilam, Iran;; 2 Non-Communicable Diseases Center, Ilam University of Medical Sciences, Ilam, Iran

**Keywords:** Anemia, COVID-19, Risk factors, Mortality, Survival

## Abstract

**Background:**

Anemia often worsens the severity of respiratory illnesses, and few studies have so far elucidated the impact of anemia on COVID-19 infection. This study aimed to evaluate the effect of anemia at admission on the overall survival of COVID-19 patients using AFT models.

**Methods:**

This registry-based, single-center retrospective cohort study was conducted in a university hospital in Ilam, the southwest of Iran, between March 2020 and September 2021. AFT models were applied to set the data of 2,441 COVID-19 patients. Performance of AFT models was assessed using AIC and Cox-Snell residual. On-admission anemia was defined as Hb concentration <120 g/l in men, <110 g/l in women, and <100 g/l in pregnant women.

**Results:**

The median in-hospital survival times for anemic and non-anemic patients were 27 and 31 days, respectively. Based on the AIC and Cox-Snell residual graph, the Weibull model had the lowest AIC and it was the best fitted model to the data set among AFT models. In the adjusted model, the results of the Weibull model suggested that the anemia (adjusted TR: 1.04; 95% CI: 1.00-1.08; *p* = 0.03) was the accelerated factor for progression to death in COVID-19 patients. Each unit of increase in hemoglobin in COVID-19 patients enhanced the survival rate by 4%.

**Conclusion:**

Anemia is an independent risk factor associated with the risk of mortality from COVID-19 infection. Therefore, healthcare professionals should be more sensitive to the Hb level of COVID-19 patients upon admission.

## INTRODUCTION

Despite widespread vaccination and other preventive measures, the COVID-19 pandemic has a precarious situation^[^^1^^]^. This disaster has posed serious challenges to health systems in many countries in the world^[^^2^^]^. Since the beginning of the COVID-19 pandemic, the worldwide deaths related to this disease have exceeded six million patients, and more than 100,000 patients have died in Iran. Besides, old adults and patients with underlying medical conditions such as anemia are at the high risk of death^[^^3^^]^.

 Anemia is a condition characterized by the reduction of the oxygen-carrying capacity due to decreased concentration of Hb in the blood^[^^4^^]^; it affects approximately 25% of the global population^[^^5^^]^. According to the World Health Organization definition, anemia is defined as a Hb concentration <130 g/L in men and Hb <120 g/L in women^[^^6^^]^. In population-based studies, anemia has been associated with an increased risk of all-causes mortality^[^^7^^]^. Increase in the level of laboratory factors, such as C-reactive protein, D-dimer, and LDH and decrease in serum albumin, phosphate, and Hb concentration can predict severe outcomes in patients with COVID-19^[^^8^^]^. In 50% of these patients, Hb level decreases, and anemia is one of the common manifestations of COVID-19^[^^9^^,^^10^^]^. Mechanisms such as decreased iron absorption, reduced erythropoietin production, increased hepcidin levels, and hemolysis in red blood cells are correlated with anemia in COVID-19 patients^[^^11^^,^^12^^]^. Anemia disrupts oxygen delivery to vital organs, followed by tissue hypoxia, which leads to the multiple organ dysfunctions, thereby causing severe and critical diseases and ultimately mortality^[^^12^^]^. 

Surveys investigating the effects of low Hb concentrations on COVID-19 infection have yielded mixed results. Some studies have reported that anemia does not directly influence COVID-19 mortality, and Hb concentration is the same in the deceased and surviving patients^[^^10^^,^^13^^,^^14^^]^. Other studies have reported that Hb concentration in ICU patients is low and is associated with severe and critical coronavirus disease^[^^9^^,^^15^^-^^17^^]^, which increases the patient's length of stay in the hospital^[^^9^^,^^18^^]^. A recent study on this area has found that higher ICU admission rate, mechanical ventilation, and odds of mortality are independently associated with anemia in COVID-19 patients^[^^18^^]^.

In literature, logistic regression has been introduced as the most extensively used model for the prediction of mortality in COVID-19 anemic patients, but it is not appropriate when the study involves time-to-event data analysis^[^^19^^]^. In medical statistics, Cox PH regression model is the most common approach for survival data analysis; however, this model does not directly generate survival times. In addition, Cox model relies on the assumption of PHs across different covariates. It means that the HR of an event is constant over time. If this hypothesis is violated, the interpretation of the coefficient regression will be biased^[^^20^^]^. As a result, we used the parametric AFT model for the analysis of survival data. AFT models directly estimate the effect of covariates on survival times and assume the multiplicative effects on the mean of survival time that covariates serve to accelerate or decelerate the time effect by some constant^[^^21^^,^^22^^]^. In these models, median survival times and also TR rather than HR are calculated^[^^19^^]^. The TR greater than one denotes that the effect of a covariate prolongs survival time and TR less than one denotes a shorter survival time^[^^23^^]^. In survival analysis, AFT models have widely been used to model diseases, including acute liver failure, leukemia, thalassemia, and cancers^[^^24^^,^^25^^]^. These models have also been applied to determine factors such as recovery time, hospital discharge, intubation periods, and the serial intervals of COVID-19 cases^[^^23^^,^^26^^,^^27^^]^. However, limited studies have been focused on investigating the relationship of at admission Hb levels at admission with COVID-19 mortality^[^^3^^,^^28^^]^, and this problem is still insufficiently explored. Based on our knowledge, AFT models have not been used for evaluating the relationship of anemia with COVID-19 mortality. In the present study, we attempted to fit AFT models with the lowest AIC to determine the effect of Hb levels at admission on acceleration/deceleration of the survival time of patients with COVID-19. 

## MATERIALS AND METHODS

This retrospective cohort study was conducted based on the registry data of patients with COVID-19 infection in a Mostafa Khomeini Hospital affiliated to Ilam University of Medical Sciences, the southwest of Iran, between March 5, 2020 and September 10, 2021. The time from the onset of symptoms to the recovery or death was considered as a survival time. Cox PH regression model was performed to analyze variables. The Schoenfeld Residuals test identified that two variables, Hb level and ICU admission, violated the proportionality assumption of Cox regression (Table 1). As a result, the AFT model was used as an alternative to the Cox PH model. The probability of overall survival was computed using the Kaplan-Meier estimator. The AFT models, such as exponential, Weibull, log-normal, log-logistic, and generalized gamma distributions, were used to model the survival time and to find the best model fitted to data based on AIC criteria and graphical Cox-Snell residual. Cox-Snell residual is a useful graphical method in assessing proportional and model fitness. Univariate data analysis was performed using the best distribution that provides the best fit for the data. A *p* value less than 0.2 was considered statistically significant in univariate analysis. All significant variables were entered into a multivariate model. Based on the lowest level of AIC, an AFT model fitted for distribution was selected for multivariate analysis. AFT models use the median survival ratio (TR) instead of the HR. Herein, anemia was defined as Hb <120 g/l in men, Hb <110 g/l in women, and Hb <100 g/l in pregnant women. The software used to analyze the data was STATA version 12 (STATA Corporation, College Station, TX, USA).

**Table 1:** Distribution of clinical qualitative and quantitative variables and Cox PH models of factors for mortality in patients with COVID-19 infection in univariate analysis

**Variables**	**Deaths**	**Survivals**	**Cox PH ratio ** **(95% CI)**	***p*** **value**	**PH ** **assumption **
Age (y; mean ± SE)	66.4 (0.9)	54.9 (0.9)	1.04 (1.03–1.04)	<0.001^*^	met
					
Gender (%)					met
Female	128 (38.3)	976 (46.3)	1^**^		
Male	206 (61.7)	1130 (53.7)	1.50 (1.18–1.84)	0.001^*^	
					
Body mass index (kg/m^2^; mean ± SE)	26.6 (0.3)	26.7 (0.1)	0.98 (0.96–1.01)	0.25	met
					
Cigarette smoking status (%)					met
Non-smoker	310 (93.9)	2011 (96.8)	1	-	
Ex-smoker	7 (2.1)	14 (0.7)	1.83 (0.86–3.89)	0.12	
Smoker	13 (4.0)	52 (2.5)	1.47 (0.84–2.56)	0.18	
					
Cardiovascular disease (%)					met
No	216 (64.9)	1682 (80.1)	1	^-^	
Yes	117 (35.1)	419 (19.9)	1.73 (1.38–2.18)	<0.001^*^	
					
Hypertension (%)					met
No	183 (54.9)	1494 (71.0)	1	-	
Yes	150 (45.1)	610 (29.0)	1.57 (1.26–1.96)	<0.001^*^	
					
Chronic lung diseases (%)					met
No	295 (88.3)	2013 (95.6)	1	-	
Yes	39 (11.7)	93 (4.4)	2.26 (1.62–3.15)	<0.001^*^	
					
Diabetes (%)					met
No	223 (67.0)	1652 (78.5)	1	^-^	
Yes	110 (33.0)	452 (21.5)	1.47 (1.17–1.85)	0.001^*^	
					
Autoimmune disease (%)					met
No	320 (96.1)	2055 (97.6)	1	-	
Yes	13 (3.9)	51 (2.4)	1.75 (1.00–3.05)	0.05^*^	
					
Neurologic disease (%)					met
No	301 (90.1)	2028 (96.3)	1	-	
Yes	33 (9.9)	78 (3.7)	1.94 (1.35 – 2.78)	0.001^*^	
					
Chronic kidney disease (%)					met
No	309 (92.5)	2027 (96.2)	1	-	
Yes	25 (7.5)	79 (3.8)	2.00 (1.33–3.01)	0.001^*^	
					
Malignancy (%)					met
No	305 (91.3)	2071 (98.3)	1	-	
Yes	29 (8.7)	35 (1.7)	3.01 (2.05–4.41)	<0.001^*^	
					
Intensive therapy unit (%)					Not met
No	55 (16.5)	1799 (85.5)	1	-	
Yes	279 (83.5)	306 (14.5)	11.06 (8.25–14.81)	<0.001^*^	
					
Anemia (%)					Not met
No	229 (71.1)	1769 (85.3)	-	-	-
Yes	93 (28.9)	305 (14.7)	1.86 (1.46–2.37)	<0.001^*^	
					
Hb (g/dl; mean ± SE)	12.72 (0.1)	13.46 (0.1)	0.89 (0.85-0.94)	<0.001^*^	Not met

^*^Significant; ^**^reference category; chronic lung diseases, asthma and chronic obstructive pulmonary diseases; PH assumption was checked by Schoenfeld residuals test; Not met, HR of an event is not constant over time. Hb levels <120 g/l in men, <110 g/l in women, and <100 g/l in pregnant women were considered as anemia.

**Fig. 1:**
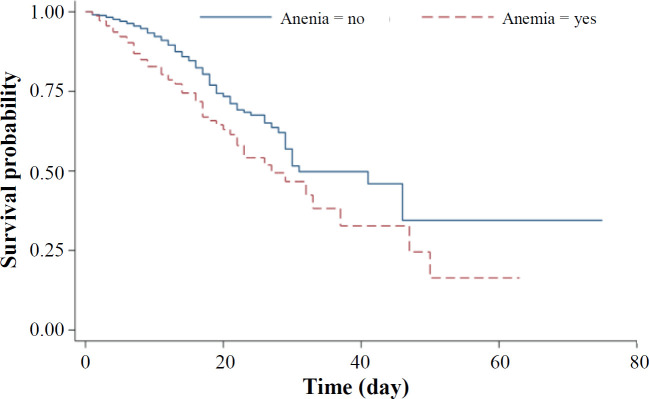
Kaplan-Meier survival curves for COVID-19 patients with and without anemia

## RESULTS

A total of 2,441 COVID-19 patients were included in the study. The mean ages of the deceased and survived patients were 66.4 ± 0.9 and 54.9 ± 0.9, respectively. The mean Hb level in the deceased patients was 12.72 ± 0.1 (ranging 3.7-18.1 g/l), while that of the survived patients was 13.46 ± 0.1 (ranging 5.1-19.4 g/l). Anemia was found in 28.9% of the deceased and 14.7% of patients who survived. Other patients' descriptive statistics are presented in Table 1. Follow-up days for all patients studied were 25,505 days; i.e. 4,303 days for the deceased and 21,202 days for the survived patients. Based on the Kaplan-Meier curve, the median survival times for the anemic and non-anemic patients were 27 and 31 days, respectively (Fig. 1). The results of Cox model showed that several variables, including Hb had a significant relationship with in-hospital mortality. However, the PH assumption was not held for Hb level, and the Cox model result was not reliable enough (Table 1). 

**Fig. 2:**
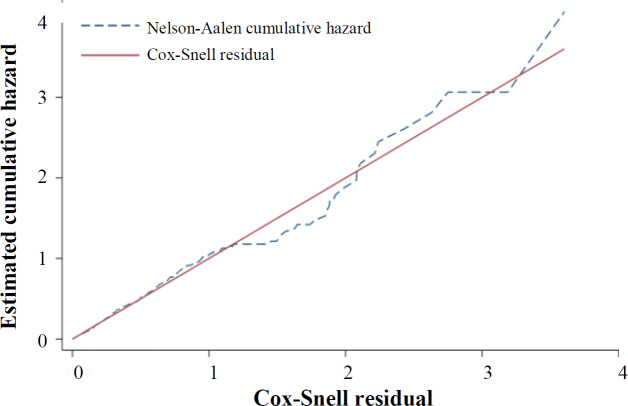
Fit of the Weibull model for time of death in COVID-19 patients by Cox-Snell residuals

**Fig. 3:**
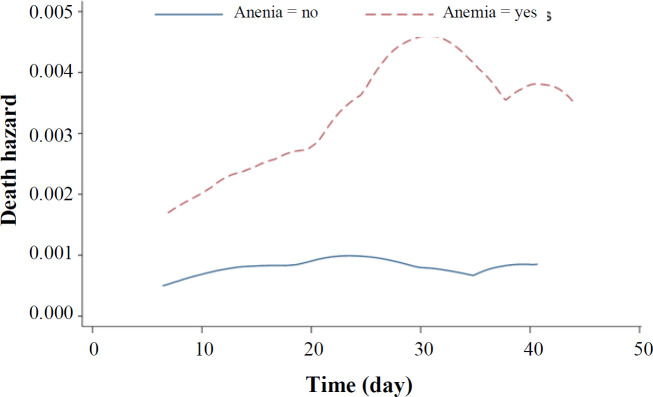
Smoothed death hazard for survival time in COVID-19 patients with and without anemia

**Fig. 4:**
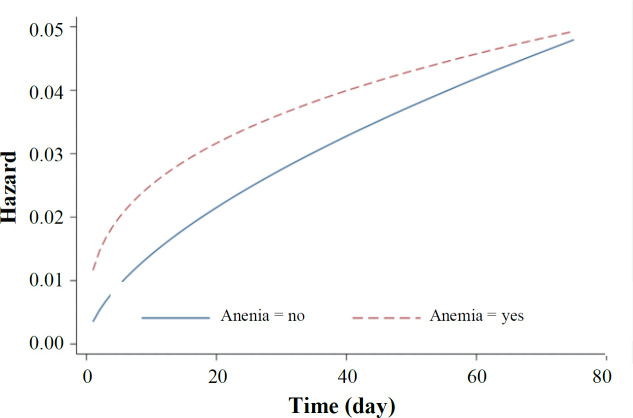
Weibull hazard function curve in patients with and without anemia

The AIC procedure was used for selection of the best AFT model fitted to the data. In univariate analysis, the Weibull distribution had the smallest AIC, and it was the best fitted model for all variables, except for cigarette smoking and Hb level. The univariate analysis of these two variables was performed with the log-logistic distribution (Table 2). The univariate analysis with log-logistic distribution confirmed a significant association between Hb level and mortality (TR: 1.09; 95% CI: 1.05-1.13; *p *< 0.001), indicating the increased survival rate with the elevation of Hb level (Table 3). In univariate analysis, variables with a *p* < 0.2 were included in the multivariate analysis. Again, the AIC and the graphical Cox-Snell residuals were used to select the best statistical distribution for the final model. The Weibull distribution had the lowest AIC, which adequately fitted to the data (Table 2). Figure 2 shows the Cox-Snell residual diagram versus the Nelson-Aalen estimator, which confirms Weibull was a best-fitting model for the data in the final multivariate analysis. The results of Weibull model showed significant effects of age, male gender, chronic lung diseases, autoimmune disease, malignancy, ICU, and Hb level on the risk of death in COVID-19 patients. This study found that high Hb level is associated with the higher survival in COVID-19 patients. In the multivariate model adjusted for other variables, the adjusted TR for Hb level was 1.04 (95% CI: 1.00-1.08; *p* = 0.03; Table 4). This finding revealed that each unit of increase in Hb has an increase in the patient's survival rate by 4%, meaning that the low level of Hb more likely develops death due to COVID-19 than normal levels. As depicted in Figure 3, COVID-19 patients with anemia had a higher risk of in-hospital death than non-anemic cases with COVID-19. The shape of the hazard function for death in COVID-19 patients with or without anemia revealed an increasing trend (Fig. 4).

## DISCUSSION

In this research, we evaluated the effects of Hb on the survival time of patients at admission, and AFT models were interpreted in terms of the speed of progression of a disease or death. Our data showed that about 16% of the hospitalized COVID-19 patients had anemia, but Faghih Dinevari *et al.*’s^[^^3^^]^ reported that on admission, 48% of COVID-19 patients were anemic. Findings of other studies demonstrated that the outbreak of anemia in hospitalized COVID-19 patients was 24.7%^[^^29^^]^. Based on the results of this study, 35% of patients with anemia were admitted to the ICU, and 18.6% underwent mechanical ventilation, while these values were 22% and 10%, respectively, for non-anemic patients. Our findings are consistent with the results of previous research that reported anemia could significantly develop poor outcomes in COVID-19 patients^[^^3^^,^^30^^,^^31^^]^. Therefore, there is a promising probability that COVID-19 patients with anemia were more likely to seize serious illnesses due to worsening lung function and poor tissue oxygenation.

**Table 2:** Discrimination among AFT models using AIC (n = 2441)

**Variables **	**Exponential**	**Weibull**	**Log** **-** **normal**	**Log** **-** **logistic**	**Generalized gamma**	**Best model**
Age	2080.8	**2007.9**	2045.1	2013.2	2009.8	Weibull
						
Gender	2180.6	**2099.2**	2140.3	2103.7	2101.1	Weibull
						
BMI	1807.6	**1721.3**	1754.9	1723.8	1723.3	Weibull
						
Cigarette smoking status	2159.9	2083.3	2118.6	**2083.2**	2084.2	Log-logistic
						
Cardiovascular disease	2156.4	**2081.1**	2116.6	2081.9	2082.9	Weibull
						
Hypertension	2162.1	**2086.6**	2123.6	2089.7	2088.6	Weibull
						
Chronic lung diseases	2171.4	**2092.4**	2128.5	2095.5	2094.4	Weibull
						
Malignancy	2164.5	**2088.9**	2126.4	2089.5	2090.7	Weibull
						
Intensive therapy unit	1769.5	**1735.1**	1815.7	1751.6	1736.3	Weibull
						
Neurologic disease	2176.8	**2101.9**	2135.8	2102.9	2103.7	Weibull
						
Autoimmune disease	2185.6	**2107.2**	2144.5	2109.8	2109.2	Weibull
						
Diabetes	2171.0	**2093.4**	2131.3	2096.9	2095.4	Weibull
						
Chronic kidney disease	2182.1	**2102.5**	2142.3	2105.9	2104.4	Weibull
						
Hb levels	2091.1	2013.5	2040.9	**2009.9**	2014.1	Log-logistic
						
Final model	1615.3	**1570.3**	1627.9	1583.6	1572.3	Weibull

The goodness of fit based on AIC criteria: -2Log-likelihood + 2P, where p is the number of parameters in the model; bold numbers: the univariate analysis for cigarette smoking and Hb level was performed with the log-logistic distribution, and for other variables, the analysis was performed using Weibull model; final model: all significant variables in univariate AFT model analysis (*p* < 0.05);

**Table 3:** Analysis of univariate AFT regression models in Hb levels and other factors for mortality from COVID-19 infection

**Variables**	**Time ratio (95% CI)**	***p*** ** value**
Age (y)	0.98 (0.97 – 0.98)	<0.001^*^
		
Gender		
Female	1^**^	
Male	0.77 (0.67 – 0.89)	0.001^*^
		
body mass index	1.01 (0.99 – 1.03)	0.31
		
Cigarette Smoking status		
Non-smoker	1	-
Ex-smoker	0.61 (0.35 – 1.09)	0.09^*^
Smoker	0.75 (0.51 – 1.11)	0.15
		
Cardiovascular disease	0.70 (0.60 – 0.81)	<0.001^*^
Hypertension	0.73 (0.63 – 0.85)	<0.001^*^
Chronic lung diseases	0.58 (0.46 – 0.72)	<0.001^*^
Diabetes	0.77 (0.66 – 0.89)	0.001^*^
Autoimmune disease	0.69 (0.48 – 0.99)	0.05^*^
Neurologic disease	0.66 (0.51 – 0.84)	0.001^*^
Chronic kidney disease	0.63 (0.48 – 0.83)	0.001^*^
Malignancy	0.49 (0.38 – 0.64)	<0.001^*^
Intensive therapy unit	0.16 (0.12 – 0.22)	<0.001^*^
Hb (gr/dl)	**1.09 (1.05 -1.13)**	**<0.001** ^*^

*Significant; ^**^reference category; TR: AFT models coefficients are expressed in exponential form (expβ), if TR > 1 associated with increased survival time and TR < 1 associated with decreased survival time; Analysis was performed with presence compared absence (reference category) for each category, except for gender, Hb, cigarette smoking, BMI, and age; Male gender was compared with reference to female gender. Cigarette Smoker and Ex-smoker were compared with reference to Non-smoker; bold number: time ratio, 95% confidence interval, and* p* value for effect of Hb on mortality.

**Table 4:** Multivariate Analysis Using Weibull AFT model for effect of Hb level on COVID-19 mortality

**Variables**	**Adjusted time ratio (95% CI)**	***p*** ** value**
Age (y)	0.98 (0.98 – 0.99)	<0.001^*^
		
Gender		
Female	1^**^	
Male	0.74 (0.62 – 0.88)	0.001^*^
		
Cigarette Smoking status		
Non-smoker	1	-
Ex-smoker	0.98 (0.57 – 1.69)	0.94
Smoker	0.80 (0.54 – 1.20)	0.28
		
Cardiovascular disease	1.01 (0.85 – 1.21)	0.91
Hypertension	0.99 (0.83 – 1.18)	0.89
Chronic lung diseases	0.70 (0.55 – 0.90)	0.006^*^
Diabetes	0.93 (0.78 – 1.11)	0.42
Autoimmune disease	0.64(0.43 – 0.96)	0.03^*^
Neurologic disease	0.86 (0.65 – 1.13	0.28
Chronic kidney disease	0.78 (0.57 – 1.06)	0.11
Malignancy	0.62 (0.46 – 0.84)	0.002^*^
Intensive therapy unit	0.21 (0.16 – 0.28)	<0.001^*^
Hb (gr/dl),	**1.04 (1.00 -1.08)**	**0.03** ^*^

*significant; ^**^reference category; analysis was performed with presence compared absence (reference category) for each category except for gender, Hb, cigarette smoking and age; Male gender was compared with reference to female gender; Cigarette smoker and Ex-smoker were compared with reference to non-smoker; bold number: time ratio, 95% confidence interval, and p value for effect of Hb on mortality.

In this study, we included 2,441 COVID-19 patients, with a median age of 66.4 ± 0.9 years. The rates of death in anemic and non-anemic COVID-19 patients were 23.4% and 11.5%, respectively. Various studies have reported different and mixed results regarding the relationship between anemia and mortality of COVID-19. These differences may be due to the types of statistical methods used, design of study, or sample size. Our study provides a further support for this relationship that low Hb level accelerated progression to death in COVID-19 patients. Some previous studies have suggested that anemia is an independent risk factor for mortality in COVID-19 patients^[^^18^^,^^28^^]^. In COVID-19 anemic patients, the insufficient Hb level reduces the oxygen-carrying capacity of blood, which leads to anoxia in tissues and worsens pneumonia^[^^32^^]^. Anemia generally intensifies the severity of respiratory illnesses, which has been shown to be accompanied by poor outcomes and possibility of developed severe pneumonia^[^^12^^, ^^33^^, ^^34^^]^. Anemia is a risk factor for longer hospital stays and other lung diseases such as chronic obstructive pulmonary disease^[^^35^^]^. Young *et al.*^[^^30^^]^ and Cecconi *et al.*^[^^36^^]^ reported no significant relationship between low hemoglobin level and the survival of COVID-19 patients. These observed differences are likely related to inclusion criteria, sample size, and study design. The association between anemia and poor outcomes in unadjusted models can be partly related to higher age and higher prevalence of some comorbidities in anemic patients. In this study, 73.4% of the anemic patients had at least one underlying health conditions. In addition, we found a significant association between the presence of comorbidities and mortality, which may be more common in anemic patients. This significant association is partly due to the effects of anemia on immunity, which in turn increases the likelihood of poor outcomes in COVID19 patients. Our results showed low Hb concentration was associated with lower survival rate in COVID-19 patients, which is inconsistent with several previous studies indicating no significant association between COVID-19 mortality and low Hb level^[^^10^^,^^17^^,^^36^^]^. These results significantly differ from the findings of our findings are in line with those previously reported in the literature^[^^18^^,^^28^^]^. 

There are some limitations in this study. First, diagnosis of anemia was conducted based on on-admission Hb level. Second, this is a single-center study, which might limit the generalizability of the results. The strengths of the present study include the large sample of COVID-19 patients, the prospective nature of the study, and the consideration of a large number of confounding factors that may affect the association between anemia and COVID-19 outcomes.

In conclusion, COVID-19 patients with anemia were more likely to develop mortality; therefore, these cases need timely intervention and should be supervised during hospitalization.

## DECLARATIONS

### Acknowledgments

The research team expresses gratitude to the Clinical Research Development Unit, Shahid Mostafa Khomeini Hospital, Ilam University of Medical Sciences, Ilam, Iran for their cooperation in data collection.

### Ethical statement

The above-mentioned sampling protocols were approved by the Medical Ethics Committee of Ilam University of Medical Sciences, Ilam, Iran (ethical code: IR.MEDILAM.REC.1399.001). 

### Data availability

The raw data supporting the conclusions of this article are available from the authors upon reasonable request. 

### Author contributions

RA: conceptualization and study design; AM: data collection, data analysis, and writing original draft; ES: writing original draft; MK: data collection and data analysis; IA: data collection and data analysis; MD: data gathering and study design; SB: conceptualization, study design, and writing original draft. All authors have read and approved the final revision of manuscript.

### Conflict of interest

None declared.

### Funding/support

The Ilam University of Medical Sciences financially supported this study.
